# OUTCOMES MEASUREMENT IN EARLY-STAGE MEMORY LOSS INTERVENTIONS: A SCOPING REVIEW

**DOI:** 10.1093/geroni/igz038.2214

**Published:** 2019-11-08

**Authors:** Anita Marie Souza, Boeun Kim, Christina Miyawaki, Basia Belza, Mee Kyung Lee, Mia Vogel, Frances Chu, Yan Su

**Affiliations:** 1 University of Washington/ School of Nursing, Seattle, Washington, United States; 2 University of Houston, Houston, Texas, United States; 3 Washington University, St Louis, Washington, United States

## Abstract

Psychosocial and psychoeducational groups are widely recommended for individuals with early stage Alzheimer’s disease and related dementias (ADRD). However, measurement challenges have hindered researchers’ efforts to demonstrate the efficacy of these groups. The purpose of this scoping review was to identify common measurement tools used in interventions for individuals with early stage ADRD and to develop suggestions for future investigations. CINAHL, Embase, PsycINFO, and PubMed were searched; 102 studies were reviewed. Inclusion criteria were set to capture intervention studies that utilized quantifiable measures with participants over age 50. Eleven articles met inclusion criteria. The majority of studies (73%) employed randomized controlled trial designs. Sample sizes ranged from N=20-236. Most commonly measured outcomes included depression, self-efficacy, self-esteem, and quality of life, but there was little consensus on how to best measure these outcomes. Standardization of psychosocial assessment tools are needed for future intervention studies with early stage ADRD.

